# Of Mice and Mates: Automated Classification and Modelling of Mouse Behaviour in Groups Using a Single Model Across Cages

**DOI:** 10.1007/s11263-024-02118-3

**Published:** 2024-06-17

**Authors:** Michael P. J. Camilleri, Rasneer S. Bains, Christopher K. I. Williams

**Affiliations:** 1 School of Informatics, University of Edinburgh, Edinburgh, UK; 2 Mary Lyon Centre, MRC Harwell, Oxfordshire, UK

**Keywords:** Joint behaviour model, Mouse behaviour model, Home-cage analysis, Mouse behaviour data, Automated behaviour classification

## Abstract

Behavioural experiments often happen in specialised arenas, but this may confound the analysis. To address this issue, we provide tools to study mice in the home-cage environment, equipping biologists with the possibility to capture the temporal aspect of the individual’s behaviour and model the interaction and interdependence between cage-mates with minimal human intervention. Our main contribution is the novel Global Behaviour Model (GBM) which summarises the joint behaviour of groups of mice across cages, using a permutation matrix to match the mouse identities in each cage to the model. In support of the above, we also (a) developed the Activity Labelling Module (ALM) to automatically classify mouse behaviour from video, and (b) released two datasets, ABODe for training behaviour classifiers and IMADGE for modelling behaviour.

**Supplementary Information:**

The online version contains supplementary material available at 10.1007/s11263-024-02118-3.

## Introduction

Understanding behaviour is a key aspect of biology, psychology and social science, e.g.  for studying the effects of treatments (Alboni et al., 2017), the impact of social factors (Langrock et al., 2014) or the link with genetics (Bains et al., 2017). Biologists often turn to model organisms as stand-ins, of which mice are a popular example, on account of their similarity to humans in genetics, anatomy and physiology (Van Meer & Raber, 2005). Traditionally, biological studies on mice have taken place in carefully controlled experimental conditions (Van Meer & Raber, 2005), in which individuals are removed from their home-cage, introduced into a specific arena and their response to stimuli (e.g.  other mice) investigated: see e.g.  the work of Arakawa et al. (2014), Casarrubea et al. (2014), Jiang et al. (2019), Qiao et al. (2018), Schank (2008), Tufail et al. (2015), Wiltschko et al. (2015). This is attractive because: (a) it presents a controlled stimulus–response scenario that can be readily quantified (Bućan & Abel, 2002), *and* (b) it lends itself easier to automated means of behaviour quantification e.g.  through top-mounted cameras in a clutter-free environment (Qiao et al., 2018; Schank, 2008; Tufail et al., 2015; Wiltschko et al., 2015).

The downside of such ‘sterile’ environments is that they fail to take into account all the nuances in their behaviour (Gomez-Marin & Ghazanfar, 2019). Such stimuli-response scenarios presume a simple forward process of perception-action which is an over-simplification of their agency (Gomez-Marin & Ghazanfar, 2019). Moreover, mice are highly social creatures, and isolating them for specific experiments is stressful and may confound the analysis (Bains et al., 2016; Crawley, 2007). For these reasons, research groups, such as the International Mouse Phenotype Consortium (Brown & Moore, 2012) and TEATIME cost-action^1^ amongst others, are advocating for the long-term analysis of rodent behaviour in the home-cage. This is aided by the proliferation of home-cage monitoring systems, but is hampered by the shortage of automated means of analysis.

In this work, we tackle the problem of studying mice in the home-cage, giving biologists tools to analyse the temporal aspect of an individual’s behaviour and model the interaction between cage-mates—while minimising disruption due to human intervention. Our contributions are: (a) a novel Global Behaviour Model (GBM) for detecting patterns of behaviour in a group setting across cages, (b) the Activity Labelling Module (ALM), an automated pipeline for inferring mouse behaviours in the home-cage from video, *and* (c) two datasets, ABODe for automated activity classification and IMADGE for analysis of mouse behaviours, both of which we make publicly available.

In this paper, we first introduce the reader to the relevant literature in Sec. 2. Section 3 describes the nature of our data, including the curation of two publicly available datasets: this allows us to motivate the methods which are detailed in Sect. 4. We continue by describing the experiments during model fitting and evaluation in Sect. 5 and conclude with a discussion of future work (Sect. 6).

## Related Work

### Experimental Setups

Animal behaviour has typically been studied over short periods in specially designated arenas—see e.g.    Arakawa et al. (2014), Casarrubea et al. (2014), Schank (2008)—and under specific stimulus–response conditions (Qiao et al., 2018). This simplifies data collection, but may impact behaviour (Bailoo et al., 2018) and is not suited to the kind of long-term studies in which we are interested. Instead, newer research uses either an enriched cage (Jiang et al., 2019; Le & Murari, 2019; Nado, 2016; Sourioux et al., 2018) or, as in our case, the home-cage itself (Bains et al., 2016; de Chaumont et al., 2019). The significance of the use of the home-cage cannot be overstated. It allows for capturing a wider plethora of nuanced behaviours with minimal intervention and disruption to the animals, but it also presents greater challenges for the automation of the analysis, and indeed, none of the systems we surveyed perform *automated behaviour classification* for *individual* mice in a *group-housed* setting.

Concerning the number of observed individuals, single-mice experiments are often preferred as they are easier to phenotype and control (Jiang et al., 2019; Nado, 2016; Sourioux et al., 2018; Wiltschko et al., 2015). However, mice are highly social creatures and isolating them affects their behaviour (Crawley, 2007), as does handling (often requiring lengthy adjustment periods). Obviously, when modelling social dynamics, the observations must perforce include multiple individuals. Despite this, there are no automated systems that consider the behaviour of each individual in the home-cage as we do. Most research is interested in the behaviour of the group as a whole (Arakawa et al., 2014; Burgos-Artizzu et al., 2012; Jiang et al., 2021; Lorbach et al., 2019), which circumvents the need to identify the individuals. Carola et al. (2011) do model a group setting, but focus on the mother only and how it relates to its litter: similarly, the social interaction test (Arakawa et al., 2014; Segalin et al., 2021) looks at the social dynamics, but only from the point of view of a resident/intruder and in a controlled setting. While Giancardo et al. (2013), de Chaumont et al. (2019) and de Chaumont et al. (2012) do model interactions, their setup is considerably different in that (a) they use specially-built arenas (not the home-cage), (b) use a top-mounted camera (which is not possible in the home-cage) and (c) classify positional interactions (e.g.  Nose-to-Nose, Head-to-Tail etc... , based on fixed proximity/pose heuristics) and not the type of individual activity (e.g.  Feeding, Drinking, Grooming etc... ).

### Automated Behaviour Classification

Classifying animal behaviour has lagged behind that of humans, with even recent work using manual labels (Carola et al., 2011; Casarrubea et al., 2014; Loos et al., 2015). Automated methods often require heavy data engineering (Arakawa et al., 2014; Dollár et al., 2005; Jiang et al., 2019, 2021). Animal behaviour inference tends to be harder because human actions are more recognisable (Le & Murari, 2019), videos are usually less cluttered (Jiang et al., 2019) and most challenges in the human domain focus on classifying short videos rather than long-running recordings as in animal observation (Jiang et al., 2021). Another factor is the limited number of publicly available animal observation datasets that target the home-cage. Most—RatSI (Lorbach et al., 2018), MouseAcademy (Qiao et al., 2018), CRIM13 (Burgos-Artizzu et al., 2012), MARS (Segalin et al., 2021), PDMB (Jiang et al., 2021), CalMS21 (Sun et al., 2021) and MABe22 (Sun et al., 2023)—use a top-mounted camera in an open field environment: in contrast, our side-view recording of the home-cage represents a much more difficult viewpoint with significantly more clutter and occlusion. Moreover, PDMB only considers pose information, while CRIM13, MARS and CalMS21 deal exclusively with a resident-intruder setup, focusing on global interactions between the two mice rather than individual actions. We aim, by releasing ABODe (Sect. 3.3), to fill this gap.

### Modelling Mouse Behaviour

The most common form of behaviour analysis involves reporting summary statistics: e.g.  of the activity levels (Geuther et al., 2019), the total duration in each behaviour (de Chaumont et al., 2019) or the number of bouts (Segalin et al., 2021), effectively throwing away the temporal information. Even where temporal models are used as by Arakawa et al. (2014), this is purely as an aid to the behaviour classification with statistics being still reported in terms of total duration in each state (behaviour). This approach provides an incomplete picture, and one that may miss subtle differences (Rapp, 2007) between individuals/groups. Some research output does report ethograms of the activities/behaviours through time (Bains et al., 2017; Ohayon et al., 2013; Segalin et al., 2021)—and Bains et al. (2016) in particular model this through sinusoidal functions—but none of the works we surveyed consider the temporal co-occurrence of behaviours between individuals in the cage as we do. For example, in MABe22, although up to three mice are present, the four behaviour labels are a multi-label setup, which indicate whether each action is evidenced at each point in time, but not which of the mice is the actor. This limits the nature of the analysis as it cannot capture inter individual dynamics, which is where our analysis comes in.

An interesting problem that emerges in biological communities is determining whether there is evidence of different behavioural characteristics among individuals/groups (Loos et al., 2015; Carola et al., 2011; Van Meer & Raber, 2005) or across experimental conditions (Bains et al., 2016; Rapp, 2007). Within the statistics and machine learning communities, this is typically the domain of *anomaly detection* for which Chandola et al. (2009) provide an exhaustive review. This is at the core of most biological studies and takes the form of hypothesis testing for significance (Carola et al., 2011). The limiting factor is often the nature of the observations employed, with most studies based on frequency (time spent or counts) of specific behaviours (Crawley, 2007; Geuther et al., 2021). The analysis by Carola et al. (2011) uses a more holistic temporal viewpoint, albeit only on individual mice, while our models consider multiple individuals. Wiltschko et al. (2015) employ Hidden Markov Models (HMMs) to identify prototypical behaviour (which they compare across environmental and genetic conditions) but only consider pose features—body shape and velocity—and do so only for individual mice. To our knowledge, we are the first to use a global temporal model inferred across cages to flag ‘abnormalities’ in another demographic.

## Datasets

**Fig. 1:**
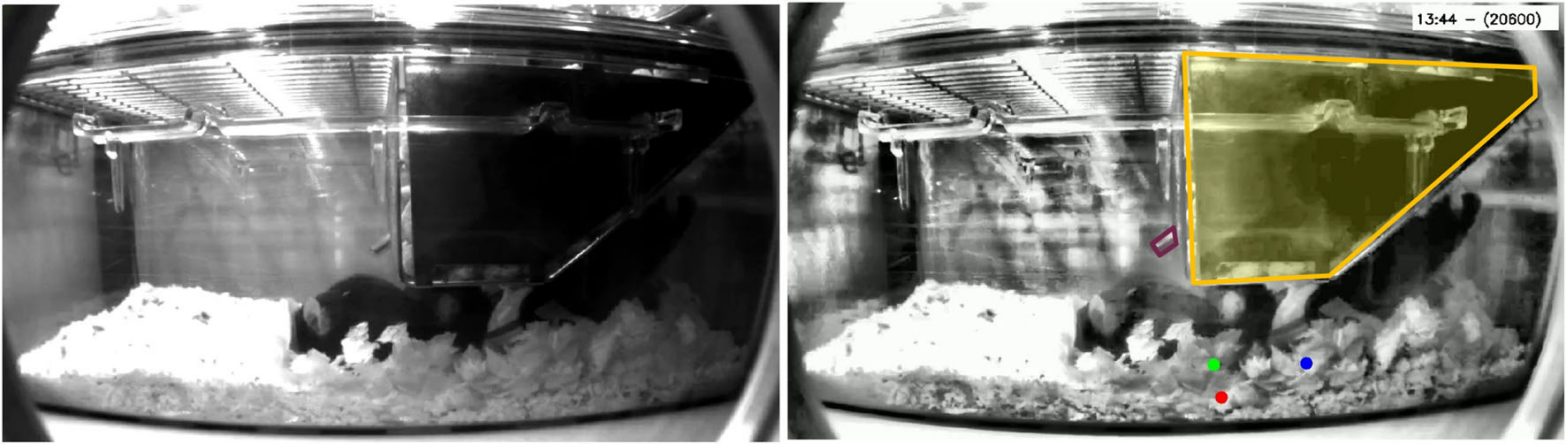
An example video frame from our data, showing the raw video (left) and an enhanced visual (right) using CLAHE (Zuiderveld, 1994). In the latter, the hopper is marked in yellow and the water spout in purple, while the (RFID) mouse positions are projected into image space and overlaid as red, green and blue dots

A key novelty of this work relates to the use of continuous recordings of *group-housed mice in the home-cage*. In line with the Reduction strategy of the 3Rs (Russell & Burch, 1959) we reuse existing data already recorded at the Mary Lyon Centre at MRC Harwell, Oxfordshire (MLC at MRC Harwell). In what follows, we describe the modalities of the data (Sec. 3.1), documenting the opportunities and challenges this presents, as well as our efforts in curating and releasing two datasets to solve the behaviour modelling (Sect. 3.2) and classification (Sect. 3.3) tasks.

### Data Sources

We use continuous three-day video and position recordings—captured using the home-cage analyses system of Bains et al. (2016)—of group-housed mice of the same sex (male) and strain (C57BL/6NTac).

#### Husbandry

The mice are housed in groups of three as a unique cage throughout their lifetime. To reduce the possibility of impacting social behaviour (Gomez-Marin & Ghazanfar, 2019), the mice have no distinguishing external visual markings: instead, they are microchipped with unique Radio-Frequency Identification (RFID) tags placed in the lower part of their abdomen. All recordings happen in the group’s own home-cage, thus minimising disruption to their life-cycle. Apart from the mice, the cage contains a food and drink hopper, bedding and a movable tunnel (enrichment structure), as shown in Fig. 1. For each cage (group of three mice), three to four day continuous recordings are performed when the mice are 3-months, 7-months, 1-year and 18-months old. During monitoring, the mice are kept on a standard 12-hour light/dark cycle with lights-on at 07:00 and lights-off at 19:00.

#### Modalities

The recordings (*video* and *position*) are split into 30-minute *segments* to be more manageable. Experiments are thus uniquely identified by the cage-id to which they pertain, the age group at which they are recorded and the segment number.

A single-channel infra-red camera captures video at 25 frames per second from a side-mounted viewpoint in $1280 \times 720$ resolution. Understandably, the hopper itself is opaque and this impacts the lighting (and ability to resolve objects) in the lower right quadrant. As regards cage elements, the hopper itself is static, and the mice can feed either from the left or right entry-points. The water-spout is on the left of the hopper towards the back of the cage from the provided viewpoint. The bedding itself consists of shavings and is highly dynamic, with the mice occasionally burrowing underneath it. Similarly, the cardboard tunnel roll can be moved around or chewed and varies in appearance throughout recordings. This clutter, together with the close confines of the cage, lead to severe occlusion, even between the mice themselves.

With no visual markings, the mice are only identifiable through the implanted RFID tag, which is picked up by a $3\times 6$ antenna-array below the cage. For visualisation purposes (and ease of reference), mice within the same cage are sorted in ascending order by their identifier and denoted Red, Green and Blue. The antennas are successively scanned in numerical order to test for the presence of a mouse: the baseplate does on average 2.5 full-scans per-second, but this is synchronised to the video frame rate. The RFID pickup itself suffers from occasional dropout, especially when the mice are above the ground (e.g.  climbing or standing on the tunnel) or in close proximity (i.e.  during huddling).

#### Identifying the Mice

A key challenge in the use of the home-cage data is the correct tracking and identification of each individual in the group. This is necessary to relate the behaviour to the individual and also to connect statistics across recordings (including different age-groups). However, the close confines of the cage and lack of visible markers make this a very challenging problem. Indeed, standard methods, including the popular DeepLabCut framework of Lauer et al. (2022) do not work on the kind of data that we use.

Our solution lies in the use of the Tracking and Identification Module (TIM), documented in Camilleri et al. (2023). We leverage Bounding Boxes (BBoxes) output by a neural network mouse detector, which are assigned to the weak location information by solving a custom covering problem. The assignment is based on a probabilistic weight model of visibility, which considers the probability of occlusion. The TIM yields per-frame identified BBoxes for the visible mice and an indication when it is not visible otherwise.

### IMADGE: A dataset for Behaviour Analysis

The Individual Mouse Activity Dataset for Group Environments (IMADGE) is our curated selection of data with the aim to provide a general dataset for analysing mouse behaviour in group settings. It includes automatically-generated localisation and behaviour labels for the mice in the cage, and is available at https://github.com/michael-camilleri/IMADGE for research use. The dataset also forms the basis for the ABODe dataset (Sec. 3.3).

#### Data Selection

IMADGE contains recordings of mice from 15 cages from the Adult (1-year) and 10 cages from the Young (3-month) age-groups: nine of the cages exist in both subsets and thus are useful for comparing behaviour dynamics longitudinally. All mice are male of the C57BL/6NTac strain. Since this strain of mice is crepuscular (mostly active at dawn/dusk), we provide segments that overlap to *any* extent with the morning (06:00–08:00) and evening (18:00–20:00) periods (at which lights are switched on or off respectively), resulting in generally $2\nicefrac {1}{2}$ hour recording *runs*. This is particularly relevant, because changes in the onset/offset of activity around these times can be very good early predictors of e.g.  neurodegenerative conditions (Bains et al., 2023). The runs are collected over the three-day recording period, yielding six runs per-cage, equivalent to 90 segments for the Adult and 61 segments for the Young age-groups.

#### Data Features

IMADGE exposes the raw video for each of the segments. The basic unit of processing for all other features, is the Behaviour Time Interval (BTI) which is one-second in duration (25 video frames). This was chosen to balance expressivity of the behaviours (reducing the probability that a BTI spans multiple behaviours) against imposing an excessive effort in annotation for training behaviour classifiers).

The main modality is the per-mouse behaviour, obtained automatically by our ALM. The observability of each mouse in each BTI is first determined: behaviour classification is then carried out on samples deemed Observable. The behaviour is according to one of seven labels: Immobile , Feeding , Drinking , Self-Grooming , Allo-Grooming , Locomotion  and Other . Behaviours are mutually exclusive within the BTI, but we retain the full probability score over all labels rather than a single class label.

The RFID-based mouse position per-BTI is summarised in two fields: the mode of the pickups within the BTI and the absolute number of antenna cross-overs. The BBoxes for each mouse are generated per-frame using our own TIM (Camilleri et al., 2023), running on each segment in turn. The per-BTI BBox is obtained by averaging the top-left/bottom-right coordinates throughout the BTI (for each mouse).

### ABODe: A dataset for Behaviour Classification

Our analysis pipeline required a mouse behaviour dataset that can be used to train models to automatically classify behaviours of interest, thus allowing us to scale behaviour analysis to larger datasets. Our answer to this need is the Annotated Behaviour and Observability Dataset (ABODe). The dataset, available at https://github.com/michael-camilleri/ABODe consists of video, per-mouse locations in the frame and per-second behaviour labels for each of the mice.

#### Data Selection

For ABODe we used a subset of data from the IMADGE dataset. We *randomly* selected 200 two-minute snippets from the Adult age-group, with 100 for Training, 40 for Validation and 60 for Testing. These were selected such that data from a cage appears exclusively in one of the splits (training/validation/test), ensuring a better estimate of generalisation performance. The data was subsequently annotated by a trained phenotyper (see appendix Sec. B).

**Fig. 2:**
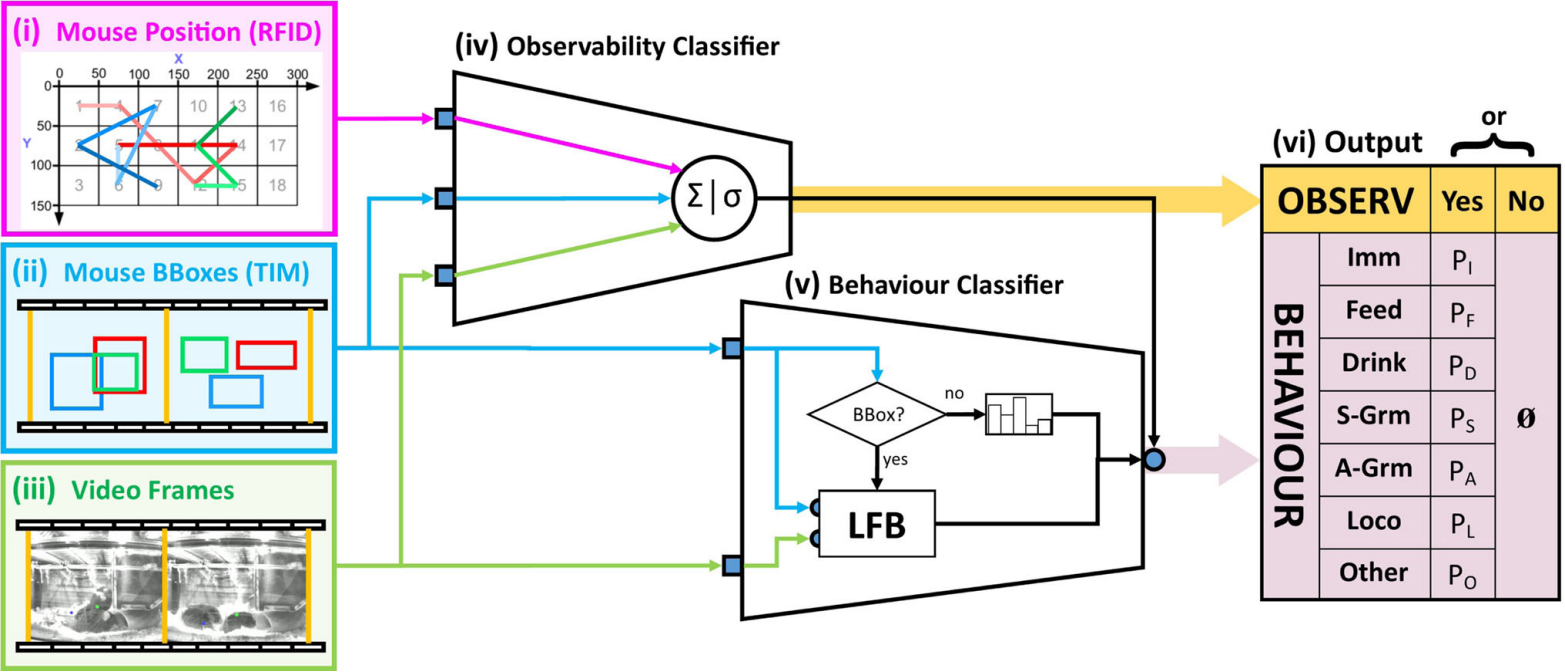
The ALM for classifying observability and behaviour *per mouse*. The input signal comes from three modalities: **i** coarse position (RFID), **ii** identified BBoxes (using the TIM as implemented in (Camilleri et al., 2023)) and **iii** video frames. An OC **iv** determines whether the mouse is observable and its behaviour can be classified. If this is the case, then the BC **v** is activated to generate a probability distribution over behaviours for the mouse. Further architectural details appear in the text

#### Data Features

As in IMADGE, ABODe contains the raw video (as two-minute snippets). We also provide the *per-frame* per-mouse RFID-based position reading and BBox location within the image (generated using TIM).

The dataset consists of 200 two-minute snippets, split as 110 Training, 30 Validation and 60 in the Test set (see Table 9). To simplify our classification and analysis, the behaviour of each mouse is defined at regular BTIs, and is either Not Observable  or one of seven mutually exclusive labels: Immobile , Feeding , Drinking , Self-Grooming , Allo-Grooming , Locomotion  or Other . We enforce that each BTI for each mouse is characterised by exactly one behaviour: this implies both exhaustibility and mutual exclusivity of behaviours. The behaviour of each mouse is annotated by a trained phenotyper  according to a more extensive labelling schema which takes into account tentative labellings and unclear annotations: this is documented in the appendix Sec. B.1. Note that unlike some other group-behaviour projects, we focus on individual actions (as above) rather than explicitly positional interactions (e.g.  Nose-to-Nose, Chasing, etc... )—this is a conscious decision that is driven by the biological processes under study as informed through years of research experience at the MLC at MRC Harwell.

## Methods

The main contribution of this work relates to the GBM which is described in Sect. 4.2. However, obtaining behaviour labels for each of the mice necessitated development of the ALM, described in Sect. 4.1.

### Classifying Behaviour: The ALM

Analysing behaviour dynamics in social settings requires knowledge of the individual behaviour throughout the observation period. Our goal is thus to label the activity of each mouse or flag that it is Not Observable  at discrete BTIs—in our case every second. A strong-point of our analysis is the volume of data we have access to: this allows our observations to carry more weight and be more relevant to the biologists as they are drawn from hours (rather than minutes) of observations. However, this scale of data is also challenging, making manual labelling infeasible.

As already argued in Secs. 2.1 and 2.2, existing setups do not consider the side-view home-cage environment that we deal with. It was thus necessary to develop our own ALM (Fig. 2), to automatically determine whether each mouse is observable in the video, and if so, infer a probability distribution over which behaviour it is exhibiting. Using discrete time-points simplifies the problem by framing it as a purely classification task, and making it easier to model (Sect. 4.2). We explicitly use a hierarchical label space (observability v. behaviour, see Fig. 2(vi)), since (a) it allows us to break down the problem using an Observability Classifier (OC) followed by a Behaviour Classifier (BC) in cascade, and (b) because we prefer to handle Not Observable  explicitly as missing data rather than having the BC infer unreliable classifications which can in turn bias the modelling. It is also semantically inconsistent to treat Not Observable  as a mutually exclusive label with the rest of the behaviours: specifically, if the mouse is Not Observable , we know it is doing exactly one of the other behaviours (even if we cannot be sure about which).

In the next subsections we describe in turn the OC and BC sub-modules: note that we postpone detailed training and experimental evidence for the choice of the architectures to our Experiments Sect. 5.

#### Determining Observability

For the OC (iv in Fig. 2) we use as features: the position of the mouse (RFID), the fraction of frames (within the BTI) in which a BBox for the mouse appears, the average area of such BBoxes and finally, the first 30 Principal Component Analysis (PCA) components from the feature-vector obtained by applying the Long-term Feature Bank (LFB) model (Wu et al., 2018) to the video. These are fed to a logistic-regression classifier trained using the binary cross-entropy loss (Bishop, 2006, 206) with $l_2$ regularisation, weighted by inverse class frequency (to address class imbalance). We judiciously choose the operating point (see Sect. 5.1.2) to balance the errors the system makes. Further details regarding the choice and training of the classifier appear in Sect. 5.1.2.

#### Probability Over Behaviours

The BC (v in Fig. 2) operates only on samples deemed Observable  by the OC, outputting a probability distribution over the seven behaviour labels (Sect. 3.3). The core component of the BC is the LFB architecture of Wu et al. (2018) which serves as the backbone activity classifier. For each BTI, the centre frame and six others on either side at a stride of eight are combined with the first detection of the mouse in the same period and fed to the LFB classifier. The logit outputs of the LFB are then calibrated using temperature scaling (Guo et al., 2017), yielding a seven-way probability vector. In instances where there is no detection for the BTI, a default distribution is output instead. All components of the BC (including choice of backbone architecture) were finetuned on our data as discussed in Sect. 5.1.3.

Although the identification of key-points on a mouse is a sensible way to extract pose information in a clean environment with a top-mounted camera, it is much more problematic in our cluttered home-cage environment with a side-mounted camera. Indeed, attempts to use the popular DeepLabCut framework (Lauer et al., 2022) failed because of the lack of reproducible key points (see previous work in Camilleri, Zhang, Bains, Zisserman, and Williams 2023, sec. 5.2). Hence we predict the behaviour with the BC directly from the RFID data, BBoxes and frames (as illustrated in Fig. 2), without this intermediate step.

**Fig. 3:**
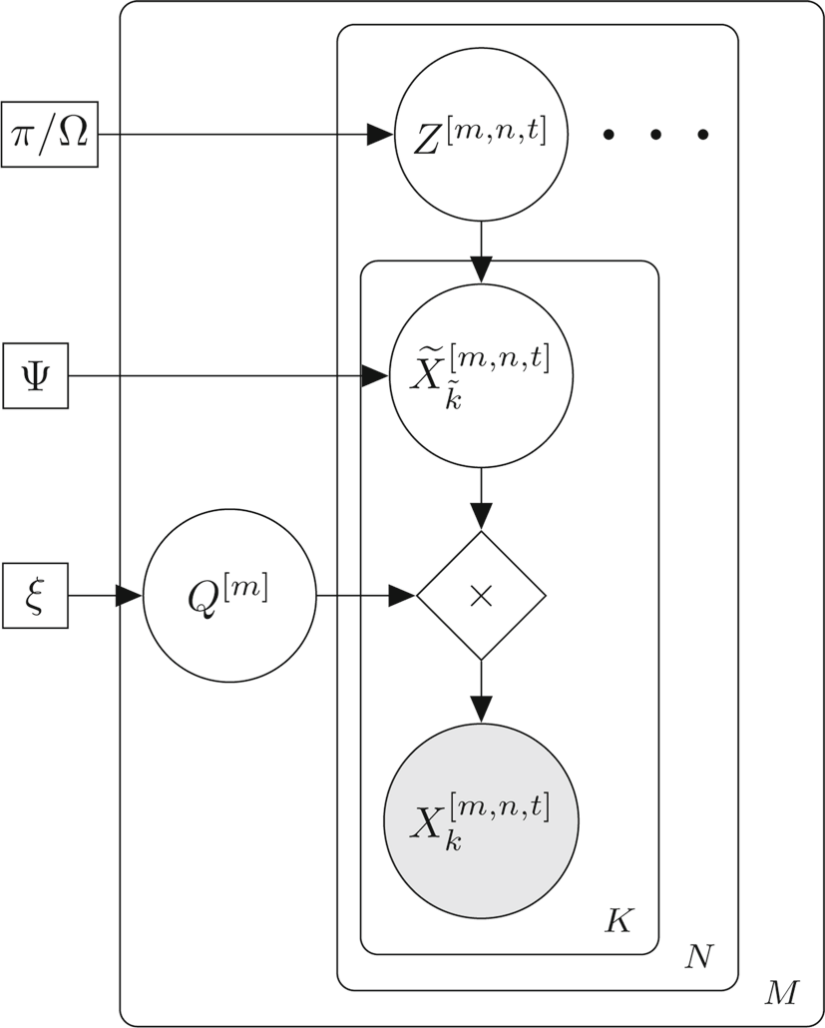
Graphical representation of our GBM. ‘$\times$’ refers to standard matrix multiplication. To reduce clutter, the model is not shown unrolled in time

### Modelling Behaviour Dynamics

In modelling behaviour, we seek to: (a) capture the temporal aspect of the individual’s behaviour, *and* (b) model the interaction and interdependence between cage-mates. These goals can be met through fitting a HMM on a per-cage basis, in which the behaviour of each mouse is represented by factorised categorical emissions contingent on a latent ‘regime’ (which couples them together). However, this generates a lot of models, making it hard to analyse and compare observations across cages.

To address this, we seek to fit one GBM across cages. The key problem is that the assignment of mouse identities in a cage (denoted as R, G, B) is arbitrary. As an example, if R represents a dominant mouse in one cage, this role may be taken by e.g.  mouse G in another cage^2^. Forcing the same emission probabilities across mice avoids this problem, but is too restrictive of the dynamics that can be modelled. Instead, we introduce a permutation matrix to match the mice in any given cage to the GBM as shown in Fig. 3. This formulation is broadly applicable to scenarios in which one seeks to uncover shared behaviour dynamics across different entities (e.g.  in the analysis of sports plays).

As in a HMM, there is a latent state *Z* indexed by cage *m*, recording-run *n* and time *t*, forming a Markov chain (over *t*), which represents the state of the cage as a whole. This ‘regime’, is parametrised by $\mathbf {\pi }$ in the first time-point (initial probability) as well as $\Omega$ (transition probabilities), and models dependence both in time as well as between individuals. We then use $\widetilde{X}$ to denote the behaviour of each mouse: this is a vector of variables, one for each mouse $\tilde{k} \in \lbrace 1, \dots , K\rbrace$, in which the order follows a ‘canonical’ assignment. Note that each mouse is represented by a complete categorical probability distribution (as output from the ALM), rather than a hard label, and is conditioned on *Z* through the emission probabilities $\Psi$. This allows us to propagate uncertainty in the outputs of the ALM module, with the error implicitly captured through $\Psi$.
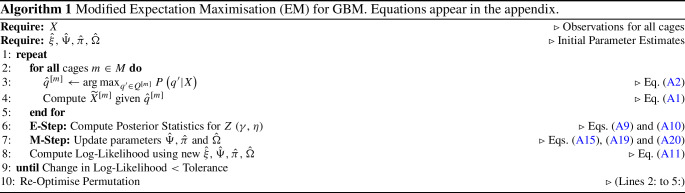


For each cage *m*, the random variable $Q^{[m]}$ governs which mouse, *k* (R/G/B) is assigned to which index, ${\tilde{k}}$, in the canonical representation ${\widetilde{X}}$, and is fixed for all samples *n*, *t* and behaviours *x*. The sample space of *Q* consists of all possible permutation matrices of size $K\times K$ i.e.  matrices whose entries are 0/1 such that there is only one ‘on’ cell per row/column. *Q* can therefore take on one of *K*! distinct values (permutations). This permutation matrix setup has been used previously, e.g.  for problems of data association in multi-target tracking (see, e.g. , Murphy 2023, sec. 29.9.3), and in static matching problems, see e.g. , Mena et al. (2020), Powell and Smith (2020) and Nazabal et al. (2023). In the above cases the focus is on approximate matching due to a combinatorial explosion, but here we are able to use exact inference due to the low dimensionality (in our case $|Q| = 3! = 6$). This is because the mouse identities have already been established though time in the TIM, and it is only the permutation of roles between cages that needs to be considered. The GBM is a novel combination of a HMM to model the interdependence between cage-mates, and the use of the permutation matrix to handle the mapping between the model’s canonical representation $\tilde{X}$ and the observed *X*.

Note that fixing *Q* and *X* determines ${\widetilde{X}}$ completely by simple linear algebra. This allows us to write out the complete data likelihood as:

1 $$\begin{aligned} P_\Theta \left( \mathcal {D}\right)&= \prod _{m, n} \Biggl ( P_\mathbf {\xi }\left( Q^{[m]}\right) P_\mathbf {\pi }\left( Z^{[.,1]}\right) \nonumber \\ {}&\times \prod _{t=2}^{T^n} P_\Omega \left( Z^{[.,t]}|Z^{[.,t-1]}\right) \nonumber \\ {}&\times \prod _{t=1}^{T^n}P_\Psi \left( X^{[.,t]}|Z^{[.,t]},Q^{[m]}\right) \Biggr ) . \end{aligned}$$

The parameters $\Theta = \left\{ \mathbf {\pi }, \Omega , \mathbf {\xi }, \Psi \right\}$ of the model are inferred through the EM algorithm (McLachlan & Krishnan, 2008) as shown in Algorithm 1 and detailed in the appendix. We seed the parameter set using a model fit to one cage, and subsequently iterate between optimising *Q* (per-cage) and optimising the remaining parameters using standard EM on the data from all cages. This procedure is carried out using models initialised by fitting to each cage in turn, and then the final model is selected based on the highest likelihood score (much like with multiple random restarts). Furthermore, we use the fact that the posterior over *Q* is highly peaked, to replace the expectation over *Q* by its maximum (a point estimate), thereby greatly reducing the computational complexity.

## Experiments

We report two sets of experiments. We begin in Sect. 5.1 by describing the optimisation of the various modules that make up the ALM, and subsequently, describe the analysis of the group behaviour in Sect. 5.2. The code to produce these results is available at https://github.com/michael-camilleri/Mice-N-Mates.

### Fine-tuning the ALM

The ALM was fit and evaluated on the ABODe dataset.

#### Metrics

For both the observability and behaviour components of the ALM we report accuracy and F$_{1}$  score (see e.g. Murphy, 2012, Sec. 5.7.2.3). We use the macro-averaged F$_{1}$  to better account for the class imbalance. This is particularly severe for the observability classification, in which only about 7% of samples are Not Observable , but it is paramount to flag these correctly. Recall that the Observable  samples will be used to infer behaviour (Sect. 4.1.2) which is in turn used to characterise the dynamics of the mice (Sect. 4.2). Hence, it is more detrimental to give False Positive (FP) outputs, which results in *Unreliable*  behaviour classifications (i.e.  when the sample is Not Observable  but the OC deems it to be Observable , which can throw the statistics awry) than missing some Observable  periods through False Negatives (FNs) (which, though *Wasteful*  of data, can generally be smoothed out by the temporal model). This construct is formalised in Table 1, where we use the terms *Unreliable*  and *Wasteful*  as they better illustrate the repercussions of the errors. In our evaluation, we report the number of *Unreliable*  and *Wasteful*  samples to take this imbalance into account. For the BC, we also report the normalised (per-sample) log-likelihood score, $\widehat{\mathcal {L}}$, given that we use it as a probabilistic classifier.

**Table 1:** Definition of classification outcomes for the Observability problem

		Predicted	
		***Obs.***	***N/Obs.***
GT	***Obs.***	True Observable [TP]	*Wasteful* [FN]
	***N/Obs.***	*Unreliable* [FP]	True Not Observable [TN]

GT refers to the ground-truth (annotated) and the standard machine learning terms—True Positive (TP), FP, True Negative (TN), FN—are in square brackets

#### Observability

**Fig. 4:**
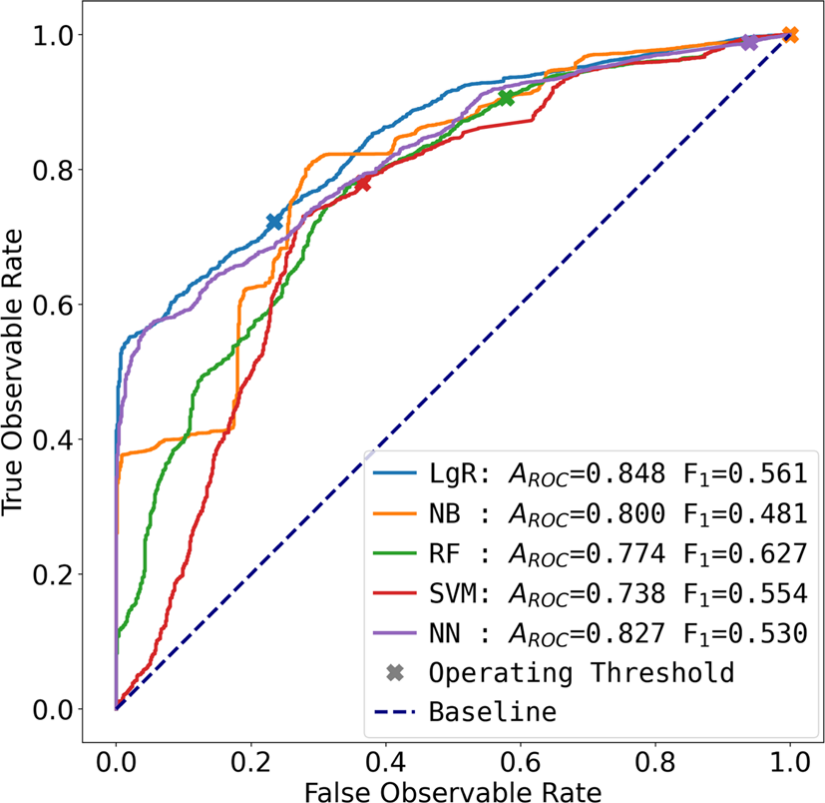
ROC curves for various architectures of the OC evaluated on the Validation split. Each coloured line shows the TP rate against the FP rate for various operating thresholds: the ‘default’ 0.5 threshold in each case is marked with a cross ‘$\times$’. The baseline (worst-case) model is shown as a dotted line

The challenge in classifying observability was to handle the severe class imbalance, which implied judicious feature selection and classifier tuning. Although the observability sample count is high within ABODe, the skewed nature (with only 7% Not Observable ) is prone to overfitting. Features were selected based on their correlation with the observability flag, and narrowed down to the subset already listed (Sect. 4.1.1). As for classifiers, we explored Logistic Regression (LgR), Naïve Bayes (NB), Random Forests (RF), Support-Vector Machines (SVM) and feed-forward Neural Networks (NN). Visualising the ROC curves (see e.g.  Murphy, 2012, Sect. 5.7.2.1) (Fig. 4), brings out two clear candidate models. Note how at most operating points, the LgR model is the best classifier, except for some ranges where NB is better (higher). These were subsequently compared in terms of the number of *Unreliable*  and *Wasteful*  samples at two thresholds: one is at the point at which the number of *Wasteful*  samples is on par with the true number of Not Observable  in the data (i.e.  8%), and the other at which the number of predicted Not Observable  equals the statistic in the ground-truth data. These appear in Table 2: the LgR outperforms the NB in almost all cases, and hence we chose the LgR classifier operating at the *Wasteful*  = 8% point.

**Table 2:** Comparison of LgR and NB as observability classifiers (on the validation set) at different operating points

	*Wasteful* = 8%		Equal Not Observable	
	*Unreliable* $\downarrow$	*Wasteful* $\downarrow$	*Unreliable* $\downarrow$	*Wasteful* $\downarrow$
LgR	**381**	**742**	499	**488**
NB	470	750	**491**	491

The best performing score in each category (column) appears in bold

Note that for context, there are 10,124 samples, of which 750 are Not Observable

#### Behaviour

We explored two architectures as backbones (feature extractors) for the BC, the Spatio-Temporal Localisation Transformer (STLT) of Radevski et al. (2021) and the LFB of Wu et al. (2018), on the basis of them being most applicable to the spatio-temporal action-localisation problem (Carreira et al., 2019). In both cases, we: (a) used pre-trained models and fine-tuned them on our data, (b) standardised the pixel intensity (over all the images) to unit mean and one standard deviation (fit on samples from the tuning split), *and* (c) investigated lighting enhancement techniques (Li et al., 2022), although this did not improve results in any of our experiments. We provide an overview of the training below, but the interested reader is directed to Camilleri (2023) for further details.

The STLT uses the relative geometry of BBoxes in a scene (layout branch), as well as the video frames (visual branch) to classify behaviour (Radevski et al. 2021). The visual branch extracts per-frame features using a ResNet-50, The layout branch is a transformer architecture which considers the temporal and spatial arrangement of detections of the subject animal and contextual objects—the other cage-mates and the hopper. In order to encode invariance to the absolute mouse identities, the assignment of the cage-mates to the slots ‘cagemate1’ and ‘cagemate2’ was randomly permuted during training. The signal from each branch is fused at the last stages of the classifier. The base architecture was extended to use information from outwith the BTI, drawing on temporal context from surrounding time-points. We ran experiments using the layout-branch only and the combined layout/visual branches, each utilising different number of frames and strides. We also experimented with focusing the visual field on the detected mouse alone. Training was done using Adam (Kingma & Ba, 2014), with random-crop and colour-jitter augmentations, and we explored various batch-sizes and learning rates: validation-set evaluation during training directed us to pick the dual-branch model with 25 frames (12 on either side of the centre frame) at a stride of 2 as the best performer.

**Table 3:** Evaluation of the baseline (prior-probability), STLT and LFB models on the Training and Validation sets in terms of Accuracy, macro-F$_{1}$  and normalised log-likelihood ($\widehat{\mathcal {L}}$)

	Train			Validate		
	Acc. $\uparrow$	F$_{1}$ $\uparrow$	$\widehat{\mathcal {L}}\uparrow$	Acc. $\uparrow$	F$_{1}$ $\uparrow$	$\widehat{\mathcal {L}}\uparrow$
baseline	0.47	0.08	$-$1.47	0.51	0.08	$-$1.45
STLT	0.77	0.45	$-$0.70	0.73	0.36	$\varvec{-1.04}$
LFB	0.96	0.93	$-$0.11	**0.74**	**0.61**	$-$2.27

The best performing score in each category (column) appears in bold

The LFB (Wu et al., 2018), on the other hand, is a dedicated spatio-temporal action localisation architecture which combines BTI-specific features with a long-term feature-bank extracted from the entire video, and joined together through an attention mechanism before being passed to a linear classification layer. Each of the two branches (feature-extractors) uses a FastRCNN network with a ResNet 50 backbone. We used the pre-trained feature-bank generator and fine-tuned the short-term branch, attention mechanism and classification layer end-to-end on our data. Training was done using Stochastic Gradient Descent (SGD) with a fixed-step learning scheduler: we used batches of 16 samples and trained for 50 epochs, varying learning rates, warm-up periods and crucially the frame sampling procedure, again in terms of total number of frames and the stride. We explored two augmentation procedures (as suggested by Wu et al. 2018): (a) random rescaling and cropping, *and* (b) colour jitter (based only on brightness and contrast). Again, validation-set scores allowed us to choose the 11-frame model with a stride of 8 and with resize-and-crop and colour-jitter augmentations (at the best performing epoch) as our LFB contender.

**Table 4:** Test performance of the ALM and baseline model, in terms of observability and behaviour

	Observability				Behaviour		
	Acc. $\uparrow$	F$_{1}$ $\uparrow$	U$\downarrow$	W$\downarrow$	Acc. $\uparrow$	F$_{1}$ $\uparrow$	$\widehat{\mathcal {L}}\uparrow$
baseline	**0.93**	0.48	1506	**0**	0.48	0.09	$-$1.47
ALM	0.88	**0.61**	**996**	1558	**0.68**	**0.54**	$\varvec{-1.06}$

The best performing score in each category (column) appears in bold

Within the former, U and W refer to the counts of *Unreliable*  and *Wasteful*  respectively: the dataset contains 20,581 samples

To choose our backbone architecture, the best performer in each case was evaluated in terms of Accuracy, F$_{1}$  and log-likelihood on both the Training and Validation set in Table 3. Given the validation set scores, the LFB model with an F$_{1}$  of 0.61 (compared to 0.36 for the STLT), was chosen as the BC. This was achieved despite the coarser layout information available to the LFB and the changes to the STLT architecture: we hypothesize that this is due to the ability of the LFB to draw on longer-term context from the whole video (as opposed to the few seconds available for the STLT).

The LFB (and even the STLT) model can only operate on samples for which there is a BBox for the mouse. We need to contend however with instances in which the mouse is not identified by the TIM, but the OC reports that it should be Observable . In this case, we fit a fixed categorical distribution to samples which exhibited this ‘error’ in the training data (i.e.  Observable  but no BBox).

#### End to End Performance

**Fig. 5:**
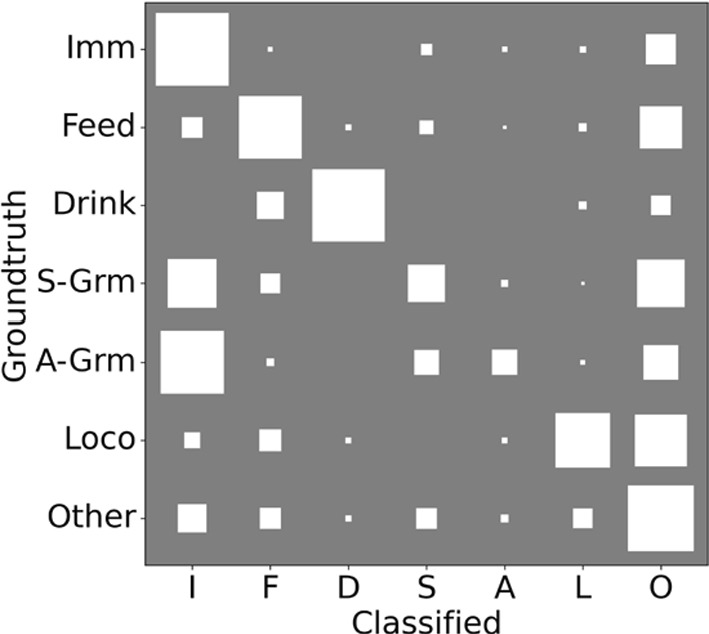
Behaviour confusion matrix of the End-to-End model as a Hinton plot. The area of each square represents the numerical value, and each row is normalized to sum to 1

In Table 4 we show the performance of the ALM on the held-out test-set. Since there is no other system that works on our data to compare against, we report the performance of a baseline classifier which returns the prior probabilities. In terms of observability, the ALM achieves slightly less accuracy but a much higher F$_{1}$  score, as it seeks to balance the types of errors (cutting the *Unreliable*  by 34%). In terms of behaviour, when considering only Observable  classifications, the system achieves 68% accuracy and 0.54 F$_{1}$  despite the high class imbalance. The main culprits for the low score are the grooming behaviours, which as shown in Fig. 5, are often confused for Immobile . Within the supplementary material, we provide a demo video – Online Resource 1–showing the output from the ALM for a sample 1:00 clip. In the clip, the mice exhibit a range of behaviours, including Feeding , Locomotion , Self-Grooming , and Immobile .

### Group Behaviour Analysis

The IMADGE dataset is used for our behaviour analysis, focusing on the adult demographic and comparing with the young one later.

#### Overall Statistics

It is instructive to look at the overall statistics of behaviours. In Table 5 we report the percentage of time mice exhibit a particular behaviour, averaged over cages and split by age-group. The Immobile  behaviour clearly stands out as the most prevalent, but there is a marked increase as the mice get older (from 30% to 45%)—this is balanced by a decrease in Other , with most other behaviours exhibiting much the same statistics.

**Table 5:** Distribution of Behaviours across cages per age-group

	Young		Adult	
	Mean (%)	Std (%)	Mean (%)	Std (%)
Imm	30.4	10.8	44.9	9.5
Feed	7.1	1.6	8.1	1.4
Drink	0.6	0.2	0.7	0.4
S-Grm	4.5	3.0	4.7	3.1
A-Grm	0.6	0.5	1.1	1.0
Loco	4.1	1.2	2.1	0.8
Other	38.5	10.2	25.9	4.1

#### Metrics

Evaluating an unsupervised model like the GBM is not straightforward, since there is no objective ground-truth for the latent states. Instead, we compare models using the normalised log-likelihood $\widehat{\mathcal {L}}$. When reporting relative changes in $\widehat{\mathcal {L}}$, we use a baseline model to set an artificial zero (otherwise the log-likelihood is not bounded from below). Let ${\widehat{\mathcal {L}}_{BL}}$ represent the normalised log-likelihood of a baseline model: the independent distribution per mouse per frame. Subsequently, we use ${\widehat{\mathcal {L}}_{\Theta }}$ for the likelihood under the global model (parametrised by $\Theta$) and ${\widehat{\mathcal {L}}_{\Theta }^*}$ for the likelihood under the per-cage model ($\Theta ^*$). We can then define the RDL between the two models parametrised as:

2 $$\begin{aligned} \text {RDL}\left( \Theta ;\Theta ^*\right) = \frac{\widehat{\mathcal {L}}_{\Theta } - \widehat{\mathcal {L}}_{\Theta ^*}}{\widehat{\mathcal {L}}_{\Theta } - \widehat{\mathcal {L}}_{BL}} \times 100 \% \quad . \end{aligned}$$

In evaluating on held-out data, we have a further complexity due to the temporal nature of the process. Specifically, each sample cannot be considered independent with respect to its neighbours. Instead, we treat each *run* ($2\nicefrac {1}{2}$ hours) as a single fold, and evaluate models using leave-one-out cross-validation: i.e.  we train on five of the folds and evaluate on the held-out in turn.

#### Size of *Z*

**Fig. 6:**
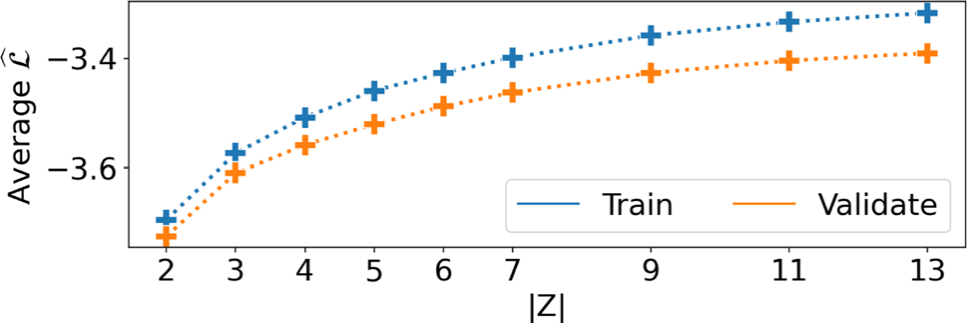
Normalised log-likelihood ($\widehat{\mathcal {L}}$) of the GBM for various dimensionalities of the latent state over all cages

The number of latent states |*Z*| in the GBM governs the expressivity of the model: too small and it is unable to capture all the dynamics, but too large and it becomes harder to interpret. To this end, we fit a per-cage model (i.e.  without the *Q* construct) to the adult mice data for varying $|Z| \in \left\{ 2, \ldots , 13 \right\}$, and computed $\widehat{\mathcal {L}}$ on held out data (using the aforementioned cross-validation approach). As shown in Fig. 6, the likelihood increased gradually, but slowed down beyond $|Z|=7$: we thus use $|Z|=7$ in our analysis.

#### Peaked Posterior Over *Q*

**Fig. 7:**
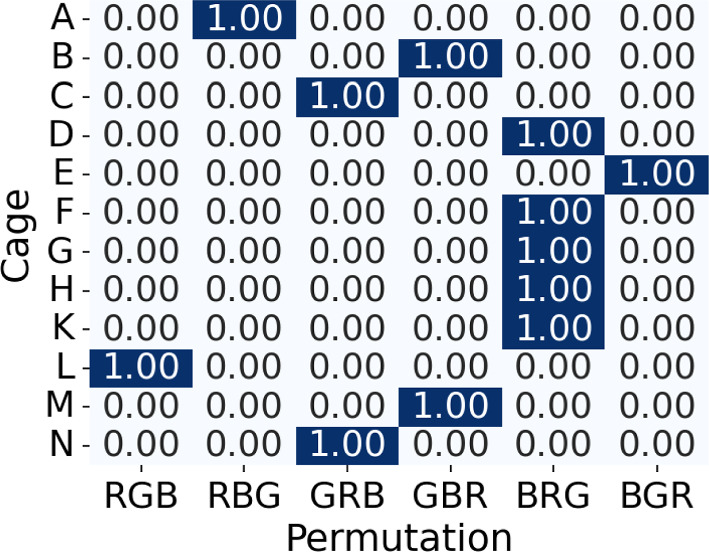
Posterior probability of the GBM over *Q* for all cages ($|Z|=7$, model trained on cage L)

Our Algorithm 1 assumes that the posterior over *Q* is sufficiently peaked. To verify this, we computed the posterior for all permutations over all cages given each per-cage model. To two decimal places, the posterior is deterministic as shown in Fig. 7 for the cages in the Adult demographic using the model trained on cage *L*. The Young demographic exhibited the same phenomenon.

#### Quality of Fit

**Fig. 8:**
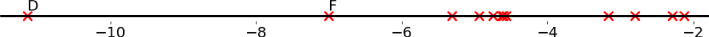
RDLs from Table 6, printed on the number line: the lowest scoring cages are marked

During training of per-cage models, we noticed extremely similar dynamics between the cages (see Camilleri 2023). This is not unexpected given that the mice are of the same strain and sex, and recorded at the same age-point. Nonetheless, we wished to investigate the penalty paid by using a global rather than per-cage model. To this end, in Table 6, we report the $\widehat{\mathcal {L}}$ and RDL (Eq. (2)) of the GBM model evaluated on data from *each cage in turn*. Although the per-cage model is understandably better than the GBM on its own data, the average drop is just 4.8%, which is a reasonable penalty to pay in exchange for a global model. Plotting the same values on the number-line (Fig. 8) shows two cages, D and F, that stand out from the rest due to a relatively higher drop. This led us to further investigate the two cages as potential outliers in our analysis, see Sect. 5.2.6.

**Table 6:** Evaluation of the GBM model ($|Z|=7$) on data from *each* cage (columns) in terms of the Normalised log-likelihood ($\widehat{\mathcal {L}}$) and RDL

	A	B	C	D	E	F	G	H	K	L	M	N
$\widehat{\mathcal {L}}_{\text {GBM}}$	$-$1.10	$-$1.17	$-$1.16	$-$1.43	$-$1.25	$-$1.36	$-$1.36	$-$1.20	$-$1.13	$-$1.22	$-$1.13	$-$1.29
RDL	$-$5.32	$-$4.56	$-$2.29	$-$11.14	$-$4.94	$-$7.00	$-$4.63	$-$3.17	$-$2.81	$-$2.13	$-$4.61	$-$4.75

#### Latent Space Analysis

**Fig. 9:**
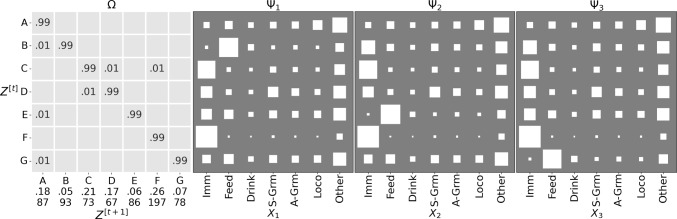
Parameters for the GBM with $|Z|=7$ trained on Adult mice. For $\Omega$ (leftmost panel) we show the transition probabilities: underneath the $Z^{[t+1]}$ labels, we also report the steady-state probabilities (first row) and the expected dwell times (in BTIs, second row). The other three panels show the emission probabilities $\Psi _k$ for each mouse as Hinton plots. We omit zeros before the decimal point and suppress values close to 0 (at the chosen precision)

**Fig. 10:**
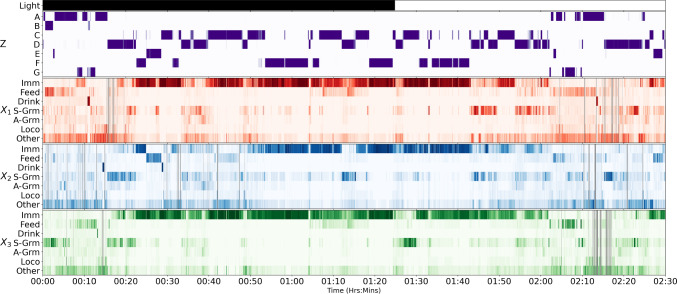
Ethogram for the regime (GBM $|Z|=7$) and individual behaviour probabilities for a run from cage B. In all but the light status, darker colours signify higher probability: the hue is purple for *Z* and matches the assignment of mice to variables $X_k$ otherwise. The light-status is indicated by white for lights-on and black for lights-off. Missing data is indicated by grey bars

Figure 9 shows the parameters of the trained GBM. Most regimes have long dwell times, as indicated by the values close to 1 on the diagonal of $\Omega$. For the emission matrices $\Psi _1, \ldots , \Psi _3$, note that regime F captures the Immobile  behaviour for all mice, and is the most prevalent (0.26 steady state probability)—it also provides evidence for the anecdotal phenomenon that mice tend to huddle together to sleep. The purity of this regime indicates that the mice often are Immobile  at the same time, re-enforcing the biological knowledge that they tend to huddle together for sleeping, but it is interesting that this was picked up by the model without any apriori bias. This is further evidenced in the ethogram visualisation in Fig. 10, which also points out regime C as indicating when any two mice are Immobile , and D for any two mice exhibiting Self-Grooming . Similarly, regime A is most closely associated with the Other  label, although it is less pure.

A point of interest are the regimes associated with the Feeding  behaviour, that are different across mice—B, E and G for mice 1, 2 and 3 respectively. This is surprising given that more than one mouse can feed at a time (the design of the hopper is such that there is no need for competition for feeding resources). This is significant, given that it is a global phenomenon, as it could be indicative of a pecking order in the cage. Another aspect that emerges is the co-occurrence of Self-Grooming  with Immobile  or Other  behaviours: note how in regime (D) (which has the highest probability of Self-Grooming ) these are the most prevalent.

**Fig. 11:**
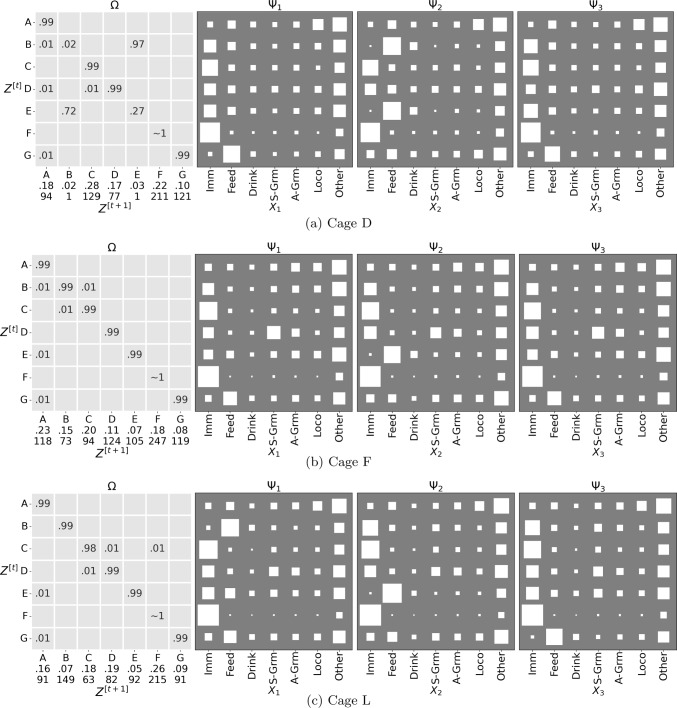
Parameters for the per-cage models ($|Z|=7$) for cages D, F and L. The order of the latent states is permuted to maximise the similarity with the global model (using the Hungarian algorithm) for easier comparison. The plot follows the arrangement in Fig. 9

Our Quality-of-Fit analysis (Sect. 5.2.5) highlighted cages D and F as exhibiting deviations in the behaviour dynamics, compared to the rest of the cages. To investigate these further, we plotted the parameters of the per-cage model for these two cases in Fig. 11, and compare them against an ‘inlier’ cage L. Note that both the latent states and the mouse identities are only identifiable subject to a permutation (this was indeed the need for the GBM construct). To make comparison easier, we optimised the permutations of both the latent states and the ordering of mice that (on a per-cage basis) maximise the agreement with the global model. The emission dynamics ($\Psi$) for the ‘outliers’ are markedly different from the global model. Note for example how the feeding dynamics for cages D and F do not exhibit the same pattern as in the GBM and cage L: in both these cases the same feeding regime G is shared by mice 1 and 3. In the case of cage D, there is also evidence that a 6-regime model suffices to explain the dynamics (note how regimes B and E are duplicates with high switching frequency between the two).

#### Anomaly Detection

**Fig. 12:**
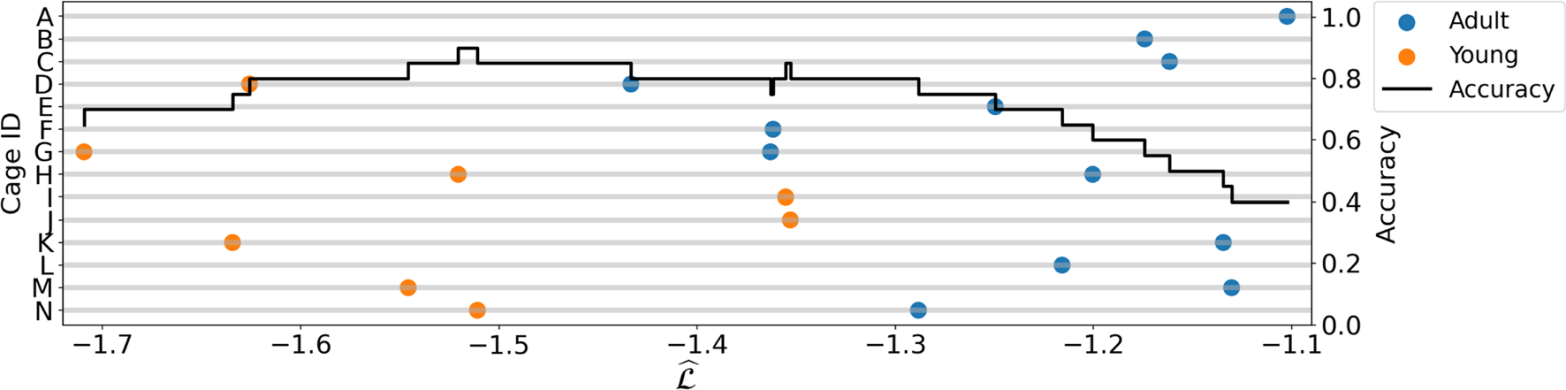
$\widehat{\mathcal {L}}$ scores (*x*-axis) of the GBM on each cage (*y*-axis, left) in the adult/young age groups, together with the accuracy of a binary threshold on the $\widehat{\mathcal {L}}$ (scale on the right)

We used the model trained on our ‘normal’ demographic to analyse data from ‘other’ cages: i.e.  anomaly detection. This capacity is useful e.g.  to identify unhealthy mice, strain-related differences, or, as in our proof of concept, evolution of behaviour through age. In Fig. 12 we show the trained GBM evaluated on data from both the adult (blue) and young (orange) demographics in IMADGE. Apart from two instances, the $\widehat{\mathcal {L}}$ is consistently lower in the younger group compared to the adult demographic: moreover, for all cages where we have data in both age groups, $\widehat{\mathcal {L}}$ is always lower for the young mice. Indeed, a binary threshold achieves 90% accuracy when optimised and a T-test on the two subsets indicates significant differences (*p*-value $=1.1\times 10^{-4}$). Given that we used mice from the same strain (indeed even from the same cages), the video recordings are very similar: consequently we expect the ALM to have similar performance on the younger demographic, suggesting that the differences arise from the behaviour dynamics.

#### Analysis of Young Mice

**Fig. 13:**
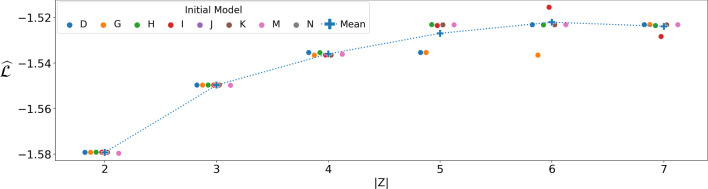
$\widehat{\mathcal {L}}$ as a function of $|Z| \in \{2, 3,..., 7\}$, with each cage as initialiser. The average (per |*Z*|) is shown as a blue cross

**Fig. 14:**
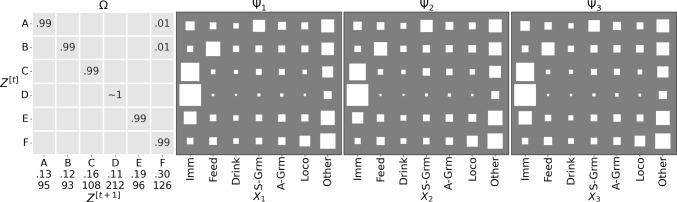
GBM parameters on the Young mice data for $|Z|=6$. Arrangement is as in Fig. 9

Training the model from scratch on the young demographic brings up interesting different patterns. Firstly, the $|Z|=6$ model emerged as a clear plateau this time, as shown in Fig. 13. Figure 14 shows the parameters for the GBM with $|Z|=6$ after optimisation on the young subset. It is noteworthy that the Immobile  state is less pronounced (in regime D), which is consistent with the younger mice being more active. Interestingly, while there is a regime associated with Feeding , it is the same for all mice and also much less pronounced: recall that for the adults, the probability of feeding was 0.7 in each of the Feeding  regimes. This could indicate that the pecking order, at least at the level of feeding, develops with age.

## Discussion

In this paper we have provided a set of tools for biologists to analyse the individual behaviours of group housed mice over extended periods of time. Our main contribution was the novel GBM—a HMM equipped with a permutation matrix for identity matching—to analyse the joint behaviour dynamics across different cages. This evidenced interesting dominance relationships, and also flagged significant deviations in an alternative young age group. In support of the above, we released two datasets, ABODe for training behaviour classifiers and IMADGE for modelling group dynamics (upon which our modelling is based). ABODe was used to develop and evaluate our proposed ALM that automatically classifies seven behaviours despite clutter and occlusion.

Since our end-goal was to get a working pipeline to allow us to model the mouse behaviour, the tuning of the ALM leaves room for further exploration, especially as regards architectures for the BC. In future work we would like to analyse other mouse demographics. Much of the pipeline should work “out of the box”, but to handle mice of different colours to those in the current dataset it may be necessary to annotate more data for the ALM and for the TIM.

Some contemporary behavioural systems use pose information to determine behaviour. Note that the STLT explicitly uses BBox poses within the attention architecture of the layout branch: nonetheless, the model was inferior to the LFB. When it comes to limb poses Camilleri, Zhang, Bains, Zisserman, and Williams (2023, sec. 5.2) showed that it is very difficult to obtain reliable pose information in our cage setup due to the level of occlusion. If however, in future work, such pose estimation can be made reliable enough in the cluttered environment of the home-cage, it could aid in improving the classification of some behaviours, such as Self-Grooming .

## Supplementary Information

Below is the link to the electronic supplementary material.

**Supplementary Material** We provide **Online Resource 1**, a demo video showing the output from the ALM system on a sample video clip. (MP4 39,426KB)

## Data Availability

The datasets used in this paper were curated by us and have been made publicly available at https://github.com/michael-camilleri/ABODe and https://github.com/michael-camilleri/IMADGE.

## Appendix A Derivations

We defined our GBM graphically in Fig. 3 and through Eq. (1) in the main text. Herein we derive the update equations for our modified EM scheme in Algorithm 1.

### A.1 Notation

We already defined our key variables in Sect. 4.2 in the main text. However, in order to facilitate our discussion, we make use of the following additional symbols. Firstly, let $\textbf{Q}^{[m]}$ represent the matrix manifestation (outcome in the sample-space) of the random variable ${Q}^{[m]}$. Secondly, we use ${\mathbb {I}^{[m,n,t]}_k}$ to signify that the observation for mouse *k* from cage *m* in sample *t* of run *n* is not available: i.e.  it is missing data. We assume that this follows a Missing-at-Random mechanism (Little & Rubin, 2002) which allows us to simply ignore such dimensions: i.e.  $\mathbb {I}$ acts as a multiplier such that it zeros out all entries corresponding to missing observations.

### A.2 Posterior Over *Q*

Due to the deterministic multiplication, selecting a particular $\textbf{Q}$, and fixing *X* (because it is observed), completely determines ${\widetilde{X}}$. Formally:

A1 $$\begin{aligned} \widetilde{X}^{[m,n,t]} = \left. \textbf{Q}^{[m]}\right. ^\top \left( X\mathbb {I}\right) ^{[m,n,t]} , \end{aligned}$$

where we have made use of the fact that for a permutation matrix, the inverse is simply the transpose. It follows that:

A2 $$\begin{aligned}&P\left( Q^{[m]} = q|X\right) \nonumber \\&\quad \propto \sum _{z',\tilde{x}'}P\left( q,X,z',\tilde{x}'\right) \nonumber \\&\quad \propto \mathbf {\xi }_q \sum _{z'}P\left( z'\right) \sum _{\tilde{x}'}P\left( \tilde{x}'|z'\right) P\left( X|\tilde{x}',q\right) \nonumber \\&\quad \propto \mathbf {\xi }_q \sum _{z'}P\left( z'\right) P\left( \widetilde{X}_{q}|z'\right) \nonumber \\&\quad = \frac{\mathbf {\xi }_q P\left( \textbf{Q}^\top \left( X\mathbb {I}\right) \right) }{\sum _{q'}\mathbf {\xi }_q' P\left( \mathbf {Q'}^\top \left( X\mathbb {I}\right) \right) } , \end{aligned}$$

where we made use of the deterministic relationship so that all probabilities over $\tilde{x}$ collapse to 0 if not following the permutation inferred by *q*. In turn, $P\left( \textbf{Q}^\top \left( X\mathbb {I}\right) \right)$ is simply the observed data likelihood of ${\widetilde{X}}$.

### A.3 Complete Likelihood and Auxiliary Function

Due to Eq. (A1), we can collapse *X* and *Q* into $\widetilde{X}$. Given that we assume the distribution over *Q* to be sufficiently peaked so that we can pick a single configuration, we can define the complete log-likelihood solely in terms of *Z* and ${\widetilde{X}}$, much like a HMM but with conditionally independent categorical emissions. Consequently, taking a Bayesian viewpoint and adding priors on each of the parameters, we define the complete data log-likelihood as:

A3 $$\begin{aligned}&P\left( \mathcal {D},\Theta |Q\right) \nonumber \\&\quad = \prod _{m,n}\left( \prod _{z=1}^{|Z|}\mathbf {\pi }_{z}^{Z_{z}^{[m,n,1]}} \prod _{t=2}^{T} \prod _{z',z} \Omega _{z',z}^{Z_{z'}^{[m,n,t-1]}Z_{z}^{[m,n,t]}} \right. \nonumber \\&\qquad \left. \prod _{t,z,k,x}\Psi _{k,z,x}^{\widetilde{X}_{k,x}^{[m,n,t]}} \right) \nonumber \\&\qquad \times \text {Dir}\left( \mathbf {\pi };\alpha ^\mathbf {\pi }\right) \prod _{z=1}^{|Z|}\text {Dir}\left( \Omega _z;\alpha ^{\Omega }_z\right) \prod _{k,z}\text {Dir}\left( \Psi _{k,z};\alpha ^{\Psi }_{k,z}\right) , \end{aligned}$$

where

$$\begin{aligned} \text {Dir}\left( \theta ;\alpha \right) = \frac{1}{\mathbb {\beta }\left( \alpha \right) }\prod _{i=1}^{|\theta |}\theta _i^{\alpha _i-1} \end{aligned}$$

is the usual Dirichlet prior with the multivariate $\beta$ normaliser function for parameter $\theta \in \left\{ \mathbf {\pi }, \Omega , \Psi \right\}$. Note that to reduce clutter, we index ${\widetilde{X}}$ using *k* and *x* rather than $\tilde{k}/\tilde{x}$.

We seek to maximise the logarithm of the above, but we lack knowledge of the latent regime *Z*. In its absence, we take the *Expectation* of the log-likelihood with respect to the latest estimate of the parameters ($\hat{\Theta }$) and the observable ${\widetilde{X}}$. We define this expectation as the ***Auxiliary*** function, $\mathcal {Q}$ (note the calligraphic form to distinguish from the permutation matrix *Q*):

A4 $$\begin{aligned}&\mathcal {Q}\left( \Theta , \hat{\Theta }\right) \equiv \ \mathbb {E}\left\langle \log \left( P\left( \mathcal {D},\Theta |Q\right) \right) |X, \Theta ^{*}\right\rangle \nonumber \\&\quad = \sum _{m,n} \Biggl ( \sum _{z=1}^{|Z|} \mathbb {E}\left\langle Z_{z}^{[m,n,1]}\right\rangle \log \left( \mathbf {\pi }_{z}\right) \nonumber \\&\qquad + \sum _{t=2}^{T} \sum _{z',z} \mathbb {E}\left\langle Z_{z'}^{[m,n,t-1]}Z_{z}^{[m,n,t]} \right\rangle \log \left( \Omega _{z',z}\right) \Biggr ) \nonumber \\&\qquad + \sum _{m,n,t,z}\mathbb {E}\left\langle Z^{[m,n,t]}_z \right\rangle \sum _{k,x}\widetilde{X}_{k,x}^{[m,n,t]}\log \left( \Psi _{k,z,x}\right) \nonumber \\&\qquad + \log \left( P\left( \Theta ;\mathcal {A}\right) \right) \end{aligned}$$

Note that the number of runs *N* can vary between cages $m \in M$, and similarly, *T* is in general different for each run *n*: however, we do not explicitly denote this to reduce clutter.

### A.4 E-Step

In Eq. (A4) have two expectations, summarised as:

A5 $$\begin{aligned} \gamma _{z}^{[m,n,t]} \equiv \mathbb {E}\left\langle Z_{z}^{[m,n,t]}\right\rangle = P\left( Z^{[m,n,t]}=z|\widetilde{X}\right) \end{aligned}$$

and

A6 $$\begin{aligned}&\eta _{z',z}^{[m,n,t]} \equiv \mathbb {E}\left\langle Z_{z'}^{[m,n,t-1]}Z_{z}^{[m,n,t]} \right\rangle \nonumber \\&\quad = P\left( Z^{[m,n,t-1]} = z', Z^{[m,n,t]} = z | \widetilde{X}\right) . \end{aligned}$$

The challenge in computing these is that it involves summing out all the other $z* \notin \left\{ z, z'\right\}$. This can be done efficiently using the recursive updates of the Baum-Welch algorithm (Baum & Petrie, 1966), which is standard for HMMs.

#### A.4.1 Recursive Updates

We first split the dependence around the point of interest *t*. To reduce clutter, we represent indexing over *m*/*n* by ‘.’ on the right hand side of equations and summarise the emission probabilities as:

$$\begin{aligned} P_{\widetilde{X}}^{[m,n]}\left( t,z\right) \equiv \left( \prod _{k=1}^K\prod _{x=1}^{|\widetilde{X}|}\Psi _{k,z,x}^{\widetilde{X}_{k,x}^{[.,t]}}\right) \end{aligned}$$

Starting with $$\gamma $$:

A7 $$\begin{aligned}&\gamma _z^{[m,n,t]} \nonumber \\&\quad = \frac{P\left( \widetilde{X}|Z^{[.,t]}_{z}\right) P\left( Z^{[.,t]}_{z}\right) }{P\left( \widetilde{X}\right) } \nonumber \\&\quad = \frac{P\left( \widetilde{X}^{[.,1:t]}|Z^{[.,t]}_{z}\right) P\left( \widetilde{X}^{[.,t+1:T]}|Z^{[.,t]}_{z}\right) P\left( Z^{[.,t]}_{z}\right) }{P\left( \widetilde{X}\right) } \nonumber \\&\quad = \frac{P\left( \widetilde{X}^{[.,1:t]},Z^{[.,t]}_{z}\right) P\left( \widetilde{X}^{[.,t+1:T]}|Z^{[.,t]}_{z}\right) }{P\left( \widetilde{X}\right) } . \end{aligned}$$

Similarly, for $$\eta $$:

A8 $$\begin{aligned}&\eta _{z',z}^{[m,n,t]} \nonumber \\&\quad = \frac{P\left( \widetilde{X} | Z^{[.,t-1]}_{z'}, Z^{[.,t]}_{z}\right) P\left( Z^{[.,t-1]}_{z'}, Z^{[.,t]}_{z}\right) }{P\left( \widetilde{X}\right) } \nonumber \\&\quad = \Biggl ( P\left( \widetilde{X}^{[.,1:t-1]} | Z^{[.,t-1]}_{z'}\right) P_{\widetilde{X}}^{[.]}\left( t,z\right) \nonumber \\&\qquad \times P\left( \widetilde{X}^{[.,t+1:T]} | Z^{[.,t]}_{z}\right) P\left( Z^{[.,t-1]}_{z'}\right) \nonumber \\&\qquad \times \Omega _{z',z} \Biggr ) \Big / P\left( \widetilde{X}\right) \nonumber \\&\quad = \Biggl ( P\left( \widetilde{X}^{[.,1:t-1]}, Z^{[.,t-1]}_{z'}\right) P_{\widetilde{X}}^{[.]}\left( t,z\right) \nonumber \\&\qquad \times P\left( \widetilde{X}^{[.,t+1:T]} | Z^{[.,t]}_{z}\right) \Omega _{z',z} \Biggr ) \Big / P\left( \widetilde{X}\right) \end{aligned}$$

We see that now we have two ‘messages’ that crucially can be defined recursively. Let the ‘forward’ pass^3^ be denoted by *F* as:

$$\begin{aligned}&F^{[m,n,t]}_{z} = P\left( \widetilde{X}^{[.,1:t]}, Z^{[.,t]}_{z}\right) \\&\quad = \sum _{z'=1}^{|Z|} \Biggl ( P\left( \widetilde{X}^{[.,1:t-1]}, Z^{[.,t-1]}_{z'}\right) P\left( Z^{[.,t]}_{z} | Z^{[.,t-1]}_{z'}\right) \\&\qquad P\left( \widetilde{X}^{[.,t]} | Z^{[.,t]}_{z}\right) \Biggr )\\&\quad = P_{\widetilde{X}}^{[.]}\left( t,z\right) \sum _{z'=1}^{|Z|} F^{[.,t-1]}_{z'} \Omega _{z',z} . \end{aligned}$$

For the special case of $t=1$, we have:

$$\begin{aligned} F^{[m,n,1]}_{z} = \mathbf {\pi }_{z} P_{\widetilde{X}}^{[.]}\left( 1,z\right) . \end{aligned}$$

Similarly, we denote the ‘backward’ recursion by *B*:

$$\begin{aligned}&B^{[m,n,t]}_{z} = P\left( \widetilde{X}^{[.,t+1:T]}|Z^{[.,t]}_{z}\right) \\&\quad = \sum _{z'=1}^{|Z|}\Biggl (P\left( Z^{[.,t+1]}_{z'}|Z^{[.,t]}_{z}\right) P\left( \widetilde{X}^{[.,t+1]}|Z^{[.,t+1]}_{z'}\right) \\&\qquad P\left( \widetilde{X}^{[.,t+2:T]}|Z^{[.,t+1]}_{z'}\right) \Biggr ) \\&\quad = \sum _{z'=1}^{|Z|} \Omega _{z,z'} P_{\widetilde{X}}^{[.]}\left( t+1,z\right) B^{[.,t+1]}_{z'} . \end{aligned}$$

Again, we have to consider the special case for $t=T$:

$$\begin{aligned} B^{[m,n,T]}_{z} = 1 . \end{aligned}$$

#### Scaling Factors

To avoid numerical underflow, we work with normalised distributions. Specifically, we define:

$$\begin{aligned} \hat{F}^{[m,n,t]}_z = P\left( Z^{[.,t]}_z|\widetilde{X}^{[.,1:t]}\right) = \frac{F^{[.,t]}_z}{P\left( \widetilde{X}^{[.,1:t]}\right) } . \end{aligned}$$

We relate these factors together through:

$$\begin{aligned} S^{[m,n,t]} = P\left( \widetilde{X}^{[.,t]}|\widetilde{X}^{[.,1:t-1]}\right) , \end{aligned}$$

and hence, from the product rule, we also have:

$$\begin{aligned} P\left( \widetilde{X}^{[m,n,1:t]}\right) = \prod _{\tau =1}^{t}S^{[.,\tau ]} . \end{aligned}$$

Consequently, we can redefine:

$$\begin{aligned} \hat{F}^{[m,n,t]}_z&= \frac{F^{[.,t]}_z}{\prod _{\tau =1}^{t}S^{[.,\tau ]}} \end{aligned}$$

and

$$\begin{aligned} \hat{B}^{[m,n,t]}_z&= \frac{B^{[.,t]}_z}{\prod _{\tau =t+1}^{T}S^{[.,\tau ]}} \end{aligned}$$

We denote for simplicity

$$\begin{aligned} C^{[m,n,t]} = \left( S^{[.,t]}\right) ^{-1} \end{aligned}$$

as the normaliser for the probability. This allows us to redefine the recursive updates for the responsibilities as follows:

A9 $$\begin{aligned} \gamma _{z}^{[m,n,t]}&= \hat{F}^{[.,t]}_{z}\hat{B}^{[.,t]}_{z} , \end{aligned}$$

and

A10 $$\begin{aligned} \eta _{z',z}^{[m,n,t]}&= C^{[.,t]}\hat{F}^{[.,t-1]}_{z'} \hat{B}_{z}^{[.,t]} \Omega _{z',z} P_{\widetilde{X}}^{[.]}\left( t,z\right) , \end{aligned}$$

where:

$$\begin{aligned} \hat{F}^{[m,n,t]}_{z}&= C^{[.,t]}\ddot{F}^{[.,t]}_{z} \end{aligned}$$

and:

$$\begin{aligned} \ddot{F}^{[m.n,t]}_{z}&= P_{\widetilde{X}}^{[.]}\left( t,z\right) \sum _{z'=1}^{|Z|} \hat{F}^{[.,t-1]}_{z'} \Omega _{z',z} \end{aligned}$$

when $$t > 1$$, and:

$$\begin{aligned} \ddot{F}^{[m.n,t]}_{z}&= P_{\widetilde{X}}^{[.]}\left( 1,z\right) \mathbf {\pi }_{z} \end{aligned}$$

if $$t = 1$$. Reformulating:

$$\begin{aligned} C^{[m,n,t]}&= \left( \sum _{z'=1}^{|Z|} \ddot{F}^{[.,t]}_{z'}\right) ^{-1} \end{aligned}$$

with:

$$\begin{aligned} \hat{B}^{[m,n,t]}_{z}&= C^{[.,t+1]}\sum _{z'} \Omega _{z,z'} P_{\widetilde{X}}^{[.]}\left( t+1,z\right) \hat{B}^{[.,t+1]}_{z'} \end{aligned}$$

for the general case ($t<T$) and:

$$\begin{aligned} \hat{B}^{[m,n,t]}_{z}&= 1 \end{aligned}$$

when $t = T$. Through the normalisers *C*, we also compute the observed data log-likelihood:

A11 $$\begin{aligned} \log \left( P\left( \widetilde{X};\Theta \right) \right) = - \sum _{m=1}^M\sum _{n=1}^N\sum _{t=1}^{T} \log \left[ C^{[.,t]}\right] . \end{aligned}$$

### A.5 M-Step

We re-arrange the $\mathcal {Q}$-function to expand and split all terms according to the parameter involved (to reduce clutter we collapse the sum over *M*/*N* and ignore constant terms):

$$\begin{aligned}&\mathcal {Q}\left( \Theta ,\hat{\Theta }\right) \\&\quad = \sum _{m,n}\sum _{z=1}^{|Z|} \gamma _{z}^{[.,1]} \log \left( \mathbf {\pi }_{z}\right) + \sum _{z=1}^{|Z|} \left( \alpha ^{\mathbf {\pi }}_{z} - 1\right) \log \left( \mathbf {\pi }_{z}\right) \\&\qquad + \sum _{m,n}\sum _{t=2}^{T} \sum _{z',z} \eta _{z',z}^{[.,t]}\log \left( \Omega _{z',z}\right) \\&\qquad + \sum _{z',z} \left( \alpha ^{\Omega }_{z',z} - 1\right) \log \left( \Omega _{z',z}\right) \\&\qquad + \sum _{m,n}\sum _{t=1}^{T}\sum _{z=1}^{|Z|}\gamma ^{[.,t]}_{z}\sum _{k=1}^K \sum _{x=1}^{|\widetilde{X}|}{\widetilde{X}^{[.,t]}_{k,x}}\log \left( \Psi _{k,z,x}\right) \\&\qquad + \sum _{z=1}^{|Z|}\sum _{k=1}^K\sum _{x=1}^{|\widetilde{X}|}\left( \alpha ^{\Psi }_{k,z,x}-1\right) \log \left( \Psi _{k,z,x} \right) \\&\qquad + Const \end{aligned}$$

#### A.5.1 Maximising for $\mathbf {\pi }$

Since we have a constraint ($\mathbf {\pi }$ must be a valid probability that sums to 1) we maximise the constrained Lagrangian:

$$\begin{aligned} \Lambda = \mathcal {Q}+ \lambda \left( \sum _{z'=1}^{|Z|}\mathbf {\pi }_{z'} - 1\right) . \end{aligned}$$

We maximise this by taking the derivative with respect to $\mathbf {\pi }_z$ and setting it to 0 (note that we can zero-out all terms involving $z' \ne z$ which are constant with respect to $\mathbf {\pi }_z$):

A12 $$\begin{aligned} \frac{\partial \Lambda }{\partial \mathbf {\pi }_z}&= \frac{1}{\mathbf {\pi }_z}\left( \sum _{m=1}^{M}\sum _{n=1}^N \gamma ^{[m,n,1]}_{z} + \alpha ^\mathbf {\pi }_z-1 \right) + \lambda = 0 \end{aligned}$$

A13 $$\begin{aligned} \lambda \mathbf {\pi }_z&= - \sum _{m=1}^M\sum _{n=1}^N \gamma ^{[m,n,1]}_{z} - \alpha ^\mathbf {\pi }_z + 1 \end{aligned}$$

Summing the above over *z*:

A14 $$\begin{aligned} \lambda&= - \sum _{m=1}^M\sum _{n=1}^N\sum _{z'=1}^{|Z|} \gamma ^{[m,n,1]}_{z'} - \sum _{z'=1}^{|Z|}\alpha ^\mathbf {\pi }_{z'} + |Z| \nonumber \\&= - \sum _{m=1}^{M}N^{m} - \sum _{z'=1}^{|Z|}\alpha ^\mathbf {\pi }_{z'} + |Z| \end{aligned}$$

In the above we have made use of the fact that both $\mathbf {\pi }_z$ and $\gamma _z^{[n]}$ sum to 1 over $z'$. Substituting Eq. (A14) for $\lambda$ in Eq. (A13) we get the maximum-a-posteriori estimate for $\hat{\mathbf {\pi }}_z$:

A15 $$\begin{aligned} \hat{\mathbf {\pi }}_z = \frac{\sum _{m=1}^M\sum _{n=1}^N\gamma ^{[m,n,1]}_{z} + \alpha ^\mathbf {\pi }_{z} - 1}{\sum _{m=1}^{M}N^{m} + \sum _{z'=1}^{|Z|}\alpha ^\mathbf {\pi }_{z'} - |Z|} . \end{aligned}$$

#### A.5.2 Maximising for $\Psi$

We follow a similar constrained optimisation procedure for $\Psi$, with the Lagrangian:

A16 $$\begin{aligned} \Lambda = \mathcal {Q}+ \sum _{k', z'}\lambda _{k',z'}\left( \sum _{x'}\Psi _{k',z',x'} - 1\right) \end{aligned}$$

Taking the derivative of Eq. (A16) with respect to $\Psi _{k,z,x}$ and setting it to 0 (ignoring constant terms):

$$\begin{aligned}&\frac{\partial \Lambda }{\partial \Psi _{k,z,x}} \\&\quad = \frac{1}{\Psi _{k,z,x}} \left( \sum _{m,n,t}\gamma ^{[m,n,t]}_{z}\widetilde{X}^{[m,n,t]}_{k,x} + \alpha ^\Psi _{k,z,x} - 1 \right) + \lambda _{k,z} \end{aligned}$$

Setting this to 0:

A17 $$\begin{aligned}&\lambda _{k, z}\Psi _{k,z,x} = - \sum _{m,n,t}\gamma ^{[m,n,t]}_{z}\widetilde{X}^{[m,n,t]}_{k,x} - \alpha ^\Psi _{k,z,x} + 1 \end{aligned}$$

Again, summing this over $x'$ yields:

A18 $$\begin{aligned} \lambda _{k,z} = - \sum _{m,n,t}\gamma ^{[m,n,t]}_{z}\sum _{x'}\widetilde{X}^{[m,n,t]}_{k,x'} - \sum _{x'}\alpha ^\Psi _{k,z,x'} + |\widetilde{X}| \end{aligned}$$

Substituting Eq. (A18) back into Eq. (A17) gives us:

A19 $$\begin{aligned} \hat{\Psi }_{k,z,x} = \frac{\sum _{m,n,t}\gamma ^{[m,n,t]}_{z}\widetilde{X}^{[m,n,t]}_{k,x} + \alpha ^\Psi _{k,z,x} - 1}{\sum _{m,n,t}\gamma ^{[m,n,t]}_{z}\sum _{x'}\widetilde{X}^{[m,n,t]}_{k,x'} + \sum _{x'}\alpha ^\Psi _{k,z,x'} - |\widetilde{X}|} . \end{aligned}$$

#### A.5.3 Maximising for $\Omega$

As always, this is a constrained optimisation by virtue of the need for valid probabilities. We start from the Lagrangian:

$$\begin{aligned} \Lambda&= \mathcal {Q}+ \sum _{z^\dagger =1}^{|Z|}\lambda _{z^\dagger }\left( \sum _{z^*=1}^{|Z|}\Omega _{z^\dagger ,z^*} - 1 \right) \\ \frac{\partial \Lambda }{\partial \Omega _{z',z}}&= \frac{1}{\Omega _{z',z}}\left( \sum _{m,n} \sum _{t=2}^{T} \eta _{z',z}^{[., t]} + \left( \alpha ^\Omega _{z',z}-1\right) \right) + \lambda _{z'} \\ \lambda _{z'}\Omega _{z',z}&= - \left( \sum _{m,n} \sum _{t=2}^{T} \eta _{z',z}^{[., t]} + \left( \alpha ^\Omega _{z',z}-1\right) \right) \\ \lambda _{z'}&= - \left( \sum _{m,n} \sum _{t=2}^{T} \sum _{z^*=1}^{|Z|}\eta _{z',z^*}^{[., t]} + \sum _{z^*=1}^{|Z|}\alpha ^\Omega _{z',z^*} - |Z| \right) \end{aligned}$$

which after incorporating into the previous equation gives the maximum-a-posteriori update:

A20 $$\begin{aligned} \hat{\Omega }_{z',z} = \frac{\sum _{m,n} \sum _{t=2}^{T} \eta _{z',z}^{[., t]} + \left( \alpha ^\Omega _{z',z}-1\right) }{\sum _{m,n} \sum _{t=2}^{T} \sum _{z^*}\eta _{z',z^*}^{[., t]} + \sum _{z^*}\alpha ^\Omega _{z',z^*} - |Z|} \end{aligned}$$

## Appendix B Labelling Behaviour in ABODe

A significant effort in the curation of the ABODe data was the annotation of the behaviours. This involved development of a well-defined annotation schema, and a rigorous annotation process, building upon the expertise of the animal care technicians at MLC at MRC Harwell and our own experience in annotation processes.

### B.1 Annotation Schema

Labels are specified per-mouse per-BTI, focusing on both the *behaviour* and also an indication of whether it is *observable*.

#### B.1.1 Behaviour

The schema admits nine behaviours and three other labels, as shown in Table 7. In particular, labels Hidden , Unidentifiable , Tentative  and Other  ensure that the annotator can specify a label in every instance, and clarify the source of any ambiguity. In this way, the labels are mutually exclusive and exhaustive. This supports our desire to ensure that each BTI is given exactly one behaviour label and eliminates ambiguity about the intention of the annotator.

**Table 7:** Definition of Behaviour Labels: these are listed in order of precedence (most important ones first)

Behaviour	Description
Hidden [Hid]	Mouse is fully (or almost fully) occluded and barely visible. Note that while the annotator may have their own intuition of what the mouse is doing (because they saw it before) they should still annotate as Hidden
Unidentifiable [N/ID]	Annotator cannot identify the mouse with certainty. Typically, there is at least another mouse which has an Unidentifiable flag
Immobile [Imm]	Mouse is not moving and static (apart from breathing), which may or may not be sleeping
Feeding [Feed]	The mouse is eating, typically with its mouth/head in the hopper: it may also be eating from the ground
Drinking [Drink]	Drinking from the water spout
Self-Grooming [S-Grm]	Mouse is grooming itself
Allo-Grooming [A-Grm]	Mouse is grooming another cage member. In this case, the annotator **must** indicate the recipient of the grooming through the Modifier field
Climbing [Climb]	All feet off the floor and also NOT on the tunnel, with the nose outside the food hopper if it is using it for support (i.e. it should not be eating as well)
Micro-motion [uMove]	A general motion activity while staying in the same place. This could include for example sniffing/looking around/rearing
Locomotion [Loco]	Moving/Running around
Tentative [Tent]	The mouse is exhibiting one of the behaviours in the schema, but the annotator is uncertain of which. If possible, the subset of behaviours that are tentative should be specified as a Modifier. In general, this is an indication that the behaviour needs to be evaluated by another annotator
Other [Other]	Mouse is doing something which is not accounted for in this schema. Certainly, aggressive behaviour will fall here, but there may be other behaviours we have not considered

The short-hand label (used in figures/tables) is in square brackets

#### B.1.2 Observability

The Hidden  label, while treated as mutually exclusive with respect to the other behaviours for the purpose of annotation, actually represents a hierarchical label space. Technically, Hidden  mice are doing any of the other behaviours, but we cannot tell which—any subsequent modelling might benefit from treating these differently. We thus sought to further specify the *observability* of the mice as a label-space in its own right as shown in Table 8.

**Table 8:** Definitions of Observability labels (shorthand label in square brackets)

**Observability**	**Description**
Not Observable [N/Obs]	Mouse is fully (or almost) occluded and not enough information to give any behaviour. When mice are huddling (and clearly immobile), a mouse is still considered Not Observable if none of it is visible
Observable [Obs]	Mouse is visible (or enough to distinguish between some behaviours). Note that it may still be difficult to identify with certainty what the mouse is doing but at a minimum can differentiate between ‘Immobile’ and other behaviours
Ambiguous [Amb]	All other cases, especially when it is borderline or when mouse cannot be identified with certainty

### B.2 Annotation Process

The annotations were carried out using the BORIS software (Friard & Gamba, 2016): this was chosen for its versatility, familiarity to the authors and open-source implementation.

#### B.2.1 Recruitment

Given the resource constraints in the project, we were only able to recruit a single expert animal care technician (henceforth the *phenotyper *) to do our annotations. This limited the scale of our dataset but on the other hand simplified the data curation process and ensured consistency throughout the dataset. In particular, it should be noted that given the difficulty of the task (which requires very specific expertise), the annotation cannot be crowdsourced. Camilleri (2023, Sec. 3.1.4) documents attempts to use behaviour annotations obtained through an online crowdsourcing platform: initial in-depth analysis of the data indicated that the labellings were not of sufficiently high quality as to be reliable for training our models. To mitigate the potential shortcomings of a single annotator, we: (a) carried out a short training phase for the phenotyper  with a set of clips that were simultaneously annotated by the phenotyper  and ourselves, (b) designed some automated sanity checks to be run on annotations (see below), *and*, (c) re-annotated the observability labels ourselves.

**Table 9:** Statistics for ABODe, showing the number of samples (BTIs-mouse pairs) for each behaviour and data partition

		Train	Validate	Test
Visibility	Observable	35297	9374	19075
	Hidden	2650	750	1506
Behaviour	Imm	19363	5455	10462
	Feed	3298	750	2314
	Drink	272	72	161
	S-Grm	2670	800	1512
	A-Grm	1278	339	550
	Loco	959	177	375
	Other	7457	1781	3701

#### B.2.2 Method

The phenotyper  was provided with the 200 two-minute clips, grouped in batches of 20 clips each (10 batches in total, 400 min of annotations). The clips were randomised and stratified such that in each batch there are 10 clips from the training split, 4 clips from the validation split and 6 from the testing split. This procedure was followed to reduce the effect of any shift in the annotation quality (as the phenotyper  saw more of the data) on the datasplits.

For each clip, the phenotyper  had access to the CLAHE-processed video and the RFID-based position information. We made the conscious decision to not use the BBox localisation from the TIM since this allows us to (a) decouple the behaviour annotations from the performance of upstream components, and (b) provides a more realistic estimate of end-to-end performance.

Although behaviour is defined per BTI, annotating in this manner is not efficient for humans: instead, the annotator was tasked with specifying intervals of the specific behaviour, defined by the start and end-point respectively. This is also the motivation behind limiting clips to two-minute snippets: these are long enough that they encompass several behaviours but are more manageable than the 30-minute segments, and also provide more variability (as it allows us to sample from more cages).

#### B.2.3 Quality Control

To train the phenotyper , we provided a batch of four (manually chosen) snippets, which were also annotated by ourselves—this enabled the phenotyper  to be inducted into using BORIS and in navigating the annotation schema, providing feedback as required. Following the annotation of each production batch, we also ran the labellings through a set of automated checks which guarded against some common errors. These were reported back to the phenotyper , although they had very limited time to act on the feedback which impacted on the resulting data quality.

The main data quality issue related to the misinterpretation of Hidden  by the phenotyper , leading to over-use of the Hidden  label. To rectify this, we undertook to re-visit the samples labelled as Hidden  and clarify the observability as per the schema in Table 8. Samples which the phenotyper  had labelled as anything other than Hidden  (except for Unidentifiable  samples which were ignored as ambiguous) were retained as Observable —we have no reason to believe that the phenotyper  reported a behaviour when it should have been Hidden . The only exception was when there was a clear misidentification of the mice, which was rectified (we had access to the entire segment which provided longer-term identity cues for ambiguous conditions). Note that our annotation relates to the observability (or otherwise): however, when converting a previously Hidden  sample to Observable , we provided a “*best-guess*” annotation of behaviour. These best-guess annotations are clearly marked, allowing us to defer to the superior expertise of the phenotyper  in differentiating between actual behaviours where this is critical (e.g.  for training models).

#### B.2.3 Statistics

We end this section by reporting dataset statistics. ABODe consists of 200 snippets, each two-minutes in length. These were split (across recording boundaries) into a Training, Validation and Test set. Table 9 shows the number of admissible mouse-BTIs that are annotated observable/hidden and the distribution of behaviours for observable samples.

## Footnotes

1 https://www.cost-teatime.org

2 Note that this is just an example for illustrative purposes: the GBM makes *no* assumption about any social relations within the cage.

## Code availability

Code to produce the above results is available at https://github.com/michael-camilleri/Mice-N-Mates. Note that the correct operation of the STLT and LFB models depends on external repositories outside our responsibility.
